# Complete Mitogenome of *Cruznema Tripartitum* Confirms Highly Conserved Gene Arrangement within Family Rhabditidae

**DOI:** 10.2478/jofnem-2022-0029

**Published:** 2022-09-24

**Authors:** Hongrui Du, Fan Guo, Yuxia Gao, Xuan Wang, Xue Qing, Hongmei Li

**Affiliations:** 1 Department of Plant Pathology, College of Plant Protection, Nanjing Agricultural University, Nanjing, 210095, China; 2 Key Laboratory of Integrated Management of Crop Disease and Pests, Ministry of Education, Nanjing Agricultural University, Nanjing, 210095, China

**Keywords:** Diplogasteridae, Genomics, Mitochondrial Genome, Nematode, Phylogeny, Rhabditina

## Abstract

Mitochondrial genomes have widely been used as molecular markers in understanding the patterns and processes of nematode evolution. The species in genus *Cruznema* are free-living bacterivores as well as parasites of crickets and mollusks. The complete mitochondrial genome of *C. tripartitum* was determined through high-throughput sequencing as the first sequenced representative of the genus *Cruznema*. The genome is comprised of 14,067 bp nucleotides, and includes 12 protein-coding, two rRNA, and 22 tRNA genes. Phylogenetic analyses based on amino acid data support *C. tripartitum* as a sister to the clade containing *Caenorhabditis elegans* and *Oscheius chongmingensis*. The analysis of gene arrangement suggested that *C. tripartitum* shares the same gene order with *O. chongmingensis*, *Litoditis marina*, *Diplocapter coronatus*, genus *Caenorhabditis*, and *Pristionchus pacificus*. Thus, the mitochondrial gene arrangement is highly conserved in the family Rhabditidae as well as some species in Diplogasteridae.

## Introduction

The nematode genus *Cruznema* Artigas, 1927 belongs to the family Rhabditidae Örley, 1880 under the order Rhabditida Chitwood, 1933. The species in the genus are either free-living bacterivores (Tahseen et al., 2012; Sultana and Pervez, 2019) or parasites of cricket (Reboredo and Camino, 1998; Camino and Achinelly, 2011) and mollusk (Grewal et al., 2011). Currently, seven valid species have been described, including *Cruznema campestre* Reboredo and Camino, 2000, *Cruznema graciliformis* (Goffart, 1935) Sudhaus, 1978, *Cruznema lincolnense* Reboredo and Camino, 1998, *Cruznema minimus* Sultana and Pervez, 2019, *Cruznema scarabaeum* (Sudhaus, 1978) Andrássy, 1983, *C. tripartitum* (von Linstow, 1906) Sudhaus, 1974, and *Cruznema velatum* Brzeski, 1989.

Inferring accurate phylogenies can provide significant insights into the patterns and mechanisms of evolution, and is thus a hot research topic that has been recurrently discussed among biologists. Within Nematoda, although a phylum-wide phylogeny has been established based on rRNA (Blaxter et al., 1998; Holterman et al., 2006; Bert et al., 2008), there are areas that do not seem amenable to resolution by this single nuclear gene. More recently, transcriptomes based phylogenome were introduced in Nematoda, and their role as a powerful tool in resolving phylogeny has been proven (Ahmed et al., 2021). However, the approach requires fresh nematodes, needs high sequencing depth, and involves substantial downstream bioinformatics work. In contrast, the complete mitochondrial genome (mitogenome) is a powerful alternative. It has multiple genes but in relatively small genome size, and is thus easier to handle for sequencing and downstream phylogeny reconstruction. Moreover, mitogenome is independent from nuclear gene, and thus can provide extra information on evolution background. Besides, the differences in gene order arrangement, base composition, and codon usage can also be used for comparative genomics (Jameson et al., 2003). Indeed, the mitogenome-based Nematoda phylogeny has yielded well-supported results for clades that were not well-resolved using other approaches (Kern et al., 2020).

Although there are over 200 nematode mitogenomes that have been sequenced, most of them are from economically important species or zooparasitic species (Kern et al., 2020), and very rarely are they from the free-living taxa. This bias in selection of taxa is partly a result of sequencing obstacles, as a majority of genomes have been acquired by long PCR together with Sanger sequencing technology (e.g., Hu et al., 2002; Kim et al., 2015, 2017). Given that mitogenome is highly variable while a closely related reference genome is often missing, a successful primer design and PCR amplification is practically difficult and time-consuming. The recent development of high-throughput approaches holds great promise for sequencing and annotating mitogenomes, but it has only been applied in a few nematodes (e.g., Ma et al., 2020; Qing et al., 2021).

In this study, through high-throughput sequencing in the Illumina platform, we determined the complete mitogenome of *Cruznema tripartitum*, which is the first representative of the genus. We further annotated the genome features, including gene content and genome architecture. These sequences were also used to infer the placement of *C. tripartitum* within the Nematoda by phylogenetic analysis.

## Materials and Methods

### Nematode extraction

The specimen of *C. tripartitum* were originally extracted from soil in a chestnut orchard in Guangzhou, China (GPS coordinates: 23°56¢44^2^N, 114°42¢15^2^E). This species is a free-living bacterivore, and the population was established through pure culture using nematode growth medium (NGM) supplemented with *Escherichia coli* OP50 and maintained at 24°C.

An approximate number of 8,000 living individual nematodes were used for genomic DNA extraction using Ezup Column Animal Genomic DNA Purification Kit (Sangon Biotech Co., Ltd., Shanghai, China) according to the manufacturer’s protocol. The DNA concentration was examined by a Qubit fluorometer (Invitrogen, Carlsbad, CA, USA) using the Qubit 1X dsDNA HS Kit (Yeasen Biotech, Shanghai, China). A total of 200 ng DNA extraction was used for sequencing.

### High-throughput sequencing and genome assembly

For library preparation, the TruSeq DNA Sample Prep Kit (Illumina Inc. San Diego, CA, USA) was used according to the manufacturer’s instructions. Library concentration and fragment size distribution were determined using the Qubit fluorometer and Bioanalyzer 2100 (Agilent Technologies, Santa Clara, CA, USA). Sequencing was conducted by Personalbio, Ltd. (Shanghai, China) using an Illumina NovaSeq platform.

For quality control and trimming, reads less than 50 bp, Q-score less than 20, or number of *n* > 3 were removed by FASTP (Chen et al., 2018). The resultant reads were mapped to the reference mitogenomes of 32 nematode species and 11 insect species using NextGenMap program (Sedlazeck et al., 2013) with a similarity threshold of 0.3. The candidate raw reads that matching at least one direction in the alignment results are extracted by SAMtools (Li et al., 2009). Reads were assembled in NOVOPlasty (Dierckxsens et al., 2017) using seed-extend algorithm. Since no *cox1* gene was available in GenBank for the genus *Cruznema*, a partial *cox1* belonging to the closely related *Pelodera strongyloides* (GenBank accession number LR700242) was used as a “seed” sequence. A total of 554,098 reads were used for the final assembly that generated a circular DNA molecule.

### Gene annotation and phylogenetic analyses

After mitogenome assembly, protein-coding genes (PCGs) and non-coding region were predicted using the invertebrate mitochondrial code (genetic code 5) on the MITOS web server (http://mitos.bioinf.uni-leipzig.de/index.py) (Bernt et al., 2013). Secondary structures of transfer RNA (tRNA) genes were predicted using MiTFi (Jühling et al., 2012) as implemented in MITOS and depicted using the web server FORNA (http://rna.tbi.univie.ac.at/forna/) (Kerpedjiev et al., 2015).

Multiple alignments of amino acid sequences of 12 PCGs were carried out individually using TranslatorX (Abascal et al., 2010) under invertebrate mitochondrial genetic code and MAFFT approach. The concatenate sequence matrix was generated using Geneious (Biomatters, Auckland, New Zealand) and 12 PCG alignments were arranged in following order: *nad6*-*nad5*-*nad4*-*nad4L*-*nad3*-*nad2*-*nad1*-*cytb*-*cox3*-*cox2*-*cox1*-*atp6*. The phylogeny placement of the newly sequenced *C. tripartitum* was inferred by maximum likelihood (ML) method together with the full mitogenomes of 36 other nematode species. An isopod parasitic mermithid nematode *Thaumamermis cosgrovei* was selected as the outgroup. The phylogeny tree was constructed using RAxML 8.2.12 (Stamatakis et al., 2014) with MtZoa protein substitution model and under a total of 1,000 bootstrap replicates. The phylogeny analysis was performed in the CIPRES Science Gateway (Miller et al., 2010).

## Results

### Mitogenome characterization

A total of *ca*.105 million reads (paired-end 150 bp) were generated and 99.04% of these reads were of high-quality (Q-score > 30). Approximately 10 GB raw data were mapped to the references, resulting in 0.53% mitochondrial reads; and these reads were subsequently used for mitogenome assembly.

**Figure 1:**
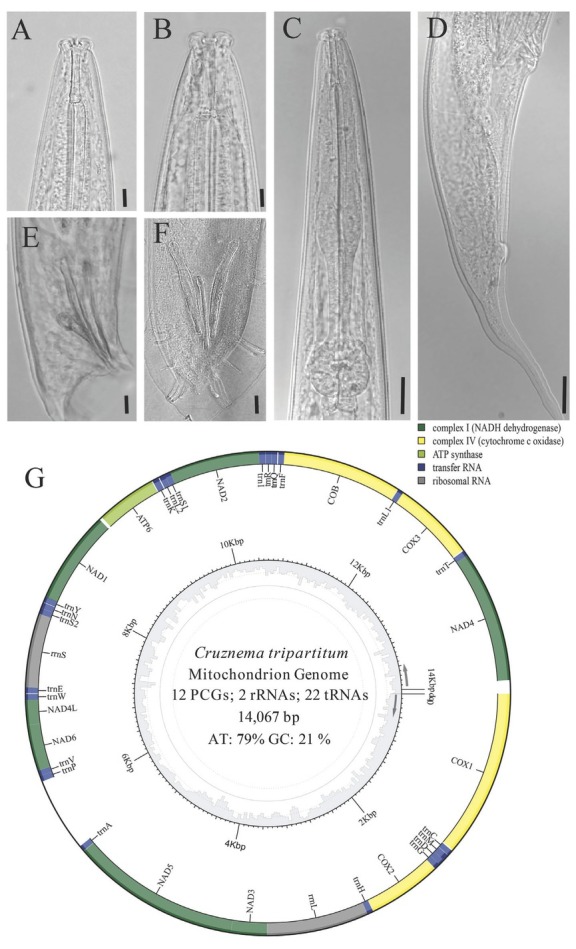
Morphology and mitogenome structure of *Cruznema tripartitum*. (A, B): Head region; (C): Anterior part of body; (D): Female tail; (E): Lateral view of male tail; (F): Ventral view of male tail; (G): Mitogenome structure. The genes are denoted by the color block and encoded in the clockwise direction. The leucine and serine tRNA genes are separated by their anticodon sequences: L1 is *trnL*-cta, L2 is *trnL*-tta, S1 is *trnS*-aga, and S2 is *trnS*-tca. The region without color indicates the non-coding region.

The complete mitogenome of *C. tripartitum* (GenBank accession OM234676) is a 14,067 bp single circular DNA that contains 12 PCGs (*atp6*, *cob*, *cox1-3*, *nad1-6*, and *nad4L*), two ribosomal RNA genes, and 22 tRNA genes (Fig. 1). The *atp8* gene was missing in the assembled mitogenome. All genes of *C. tripartitum* mitogenome are encoded in the same direction, which is a result similar to all other known chromadorean nematodes. The nucleotide composition is strongly biased toward A + T (78.7%), with 48.1% for T, 7.5% for C, 30.6% for A, and 13.8% for G. An intergenic non-coding region was found between *trnA* and *trnP* in a length of 696 bp. This region has much higher A + T content (87.1%) in comparison with any other part of mitogenome.

**Figure 2:**
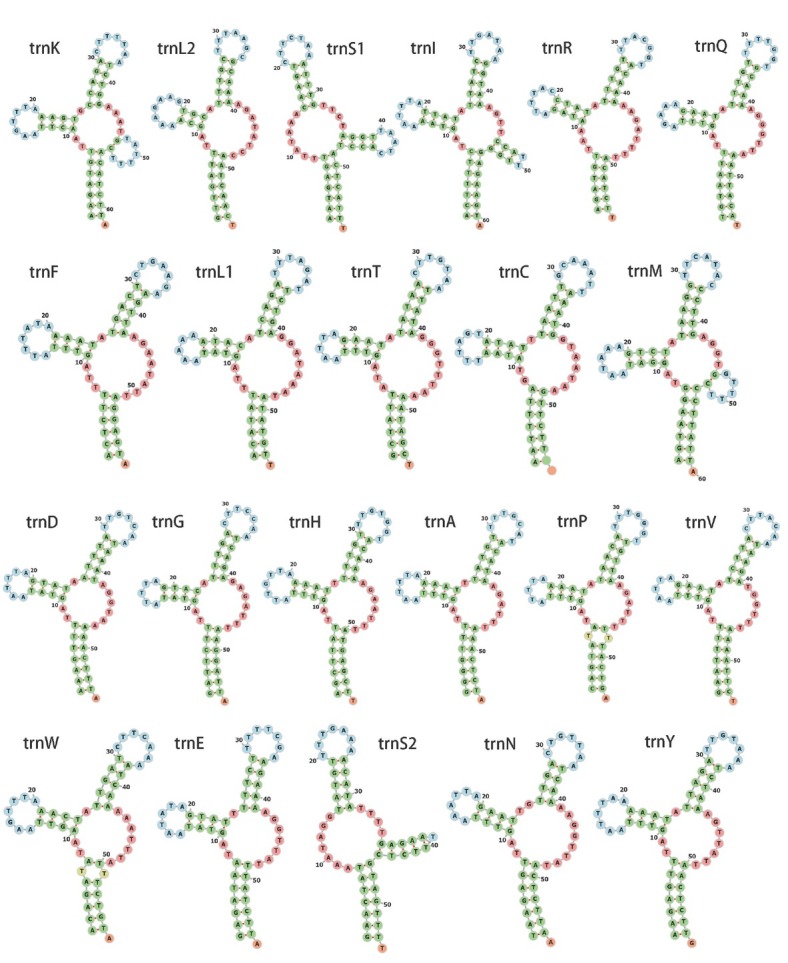
Predicted secondary structure of tRNA in the mitochondrial genome of *Cruznema tripartitum*. tRNA, transfer RNA.

For PCGs, eight genes (*cox1*, *cox2*, *cox3*, *nad2*, *nad3*, *nad5*, *nad6*, and *nad4L*) use ATT as the start codon, while another two (*atp6* and *cob*) start with ATA, and two (*nad1* and *nad4*) start with TTG (Table 1). All PCGs are preferred to use TAA as their termination codon and have T-rich codons peference. A total of 22 tRNAs were recovered, ranging in size from 54 bp (*trnK*, *trnC*, *trnP*, *trnS2*, and *trnV*) to 62 bp (*trnK*) (Table 1 and Fig. 2). Two ribosomal RNA genes are located between *trnE* and *trnS2* (*rrnS*, 697 bp) and between *trnH* and *nad3* (*rrnL*, 957 bp), respectively (Table 1). The A + T contents of *rrnS* and *rrnL* are 76.8% (36.6% A and 40.2% T) and 81.1% (37.9% A and 43.2% T), respectively.

### Phylogeny and gene arrangement

**Figure 3:**
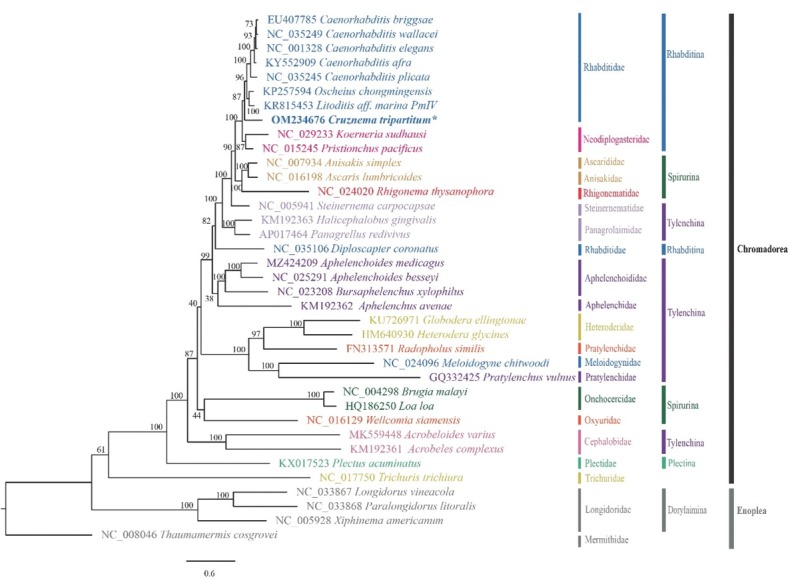
Maximum likelihood phylogenetic tree inferred from amino acid sequences of 12 concatenated protein-coding genes containing 3,893 alignment characters. The newly obtained sequence is indicated with asterisk.

Maximum likelihood phylogenetic analysis of *C. tripartitum* was conducted using the translated amino acid data set of 12 PCGs (3,893 alignment characters) together with those of 36 other nematodes (32 chromadoreans and 4 enopleans; available in NCBI GenBank) as references (Fig. 3). In general, the phylogeny we revealed was congruent with previously published phylogeny inferred by rRNA genes (Blaxter et al., 1998; De Ley and Blaxter, 2004; Holterman et al., 2006; Meldal et al., 2007) or mitogenome (Kim et al., 2015, 2017). The family Rhabditidae is paraphyletic, containing a moderately supported major clade (BS = 87) and a separated *Diploscapter coronatus* sistering to several genera in Rhabditina, Tylenchina, and Spirurina. In the major clade of Rhabditidae, the newly sequenced *C. tripartitum* is sister to *Oscheius chongmingensis*, *Litoditis marina*, and species in the genus *Caenorhabditis*.

**Figure 4:**
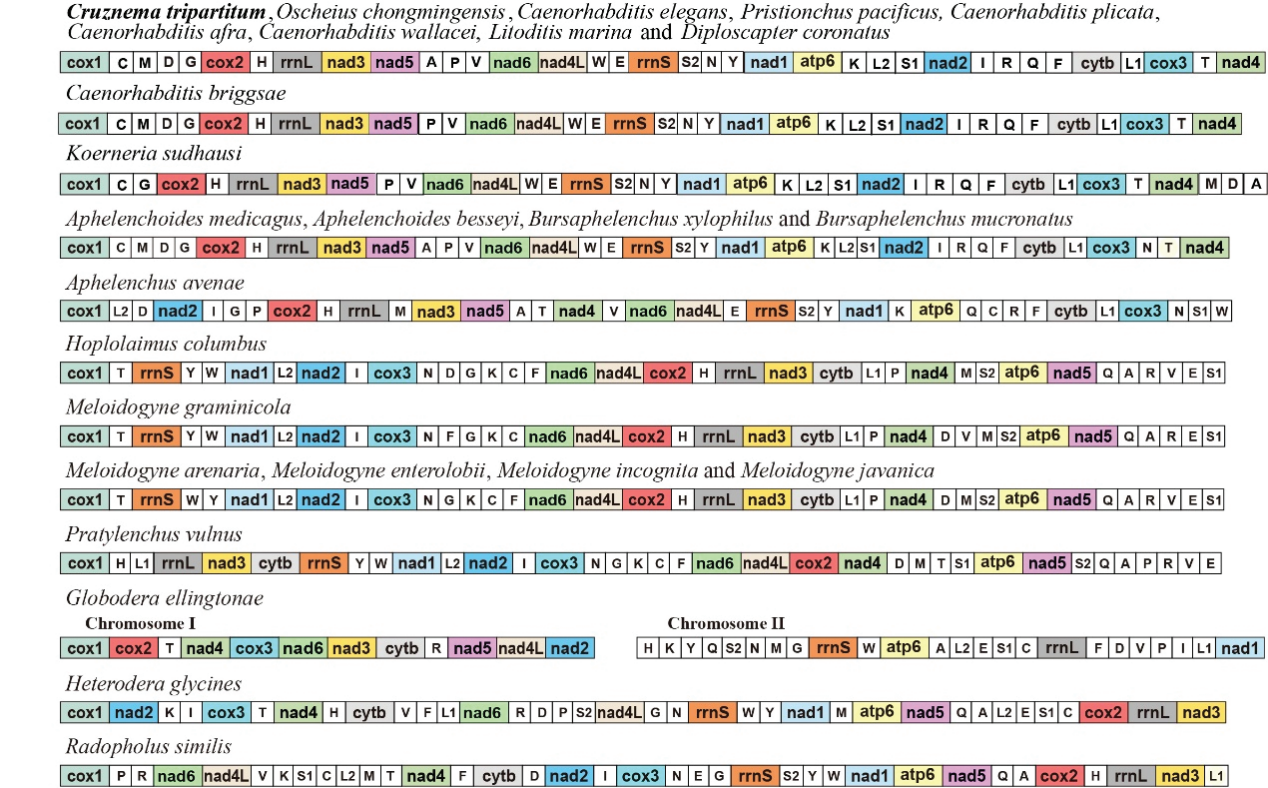
Linearized mitochondrial gene arrangement patterns of *Cruznema tripartitum* (indicated in bold) and other related species in suborders Rhabditina and Tylenchina. All genes are encoded in the same direction. All 22 tRNA genes are designated by a single letter amino acid code, based on the International Union of Pure and Applied Chemistry code. The two leucine and two serine tRNA genes are labeled according to their distinct anticodon sequences as L1 (*trnL1*-cta), L2 (*trnL2*-tta), S1 (*trnS1*-aga), and S2 (*trnS2*-tca). The non-coding regions are not included.

The analyses of gene arrangement suggested Rhabditidae representing by *C. tripartitum*, *O. chongmingensis*, *L. marina*, *Diplocapter coronatus* and genus *Caenorhabditis* are highly conserved in arrangement pattern, except for *C. briggsae* which alanine was not annotated (Fig. 4). A similar arrangement pattern was also found in some of Diplogasteridae species, i.e., *Pristionchus pacificus* shares exactly the same pattern, while *Koerneria sudhausi* only slightly differs in the placement of a few tRNAs (*trnM*, *trnD*, and *trnA*). Interestingly, such conserved arrangement may also extend to the suborder Tylenchina, e.g., the superfamily Aphelenchoidea represented by *Aphelenchoides medicagus*, *A. besseyi*, *Bursaphelenchus xylophilus*, and *B. mucronatus* share identical arrangement of PCGs to those species from Rhabditidae, although translocations can apply to some of the tRNAs.

## Discussion

The metazoan mitogenomes are approximately 14–20 kb in size and usually comprise 36–37 genes, including 12–13 PCGs (*atp6*, *atp8*, *cox1-3*, *cob*, *nad1-6*, and *nad4L*), two rRNA genes (small and large subunit ribosomal RNA), and 22 tRNA genes (Wolstenholme, 1992; Boore, 1999). The length of *C. tripartitum* mitogenome is between the range of 13,794 bp by *Caenorhabditis elegans* and 15,413 bp by *O. chongmingensis*. The assembled mitogenome consists of 12 PCGs, 22 tRNA genes, and two rRNA genes; this composition is identical to that of other reported species in Rhabditida (e.g., *C. elegans*), but different from that of the vertebrate parasite *Trichinella* (Lavrov and Brown, 2001) and many metazoans, in that the *atp8* gene is absent (Gissi et al., 2008).

**Table 1:** Mitochondrial genome organization in *Cruznema tripartitum*.

Gene name	Start	Stop	Size (bp)	Intergenic space	Codons
*COX1*	1	1575	1,575	0	ATT/TAA
*trnC* (tgc)	1587	1640	54	11	
*trnM* (atg)	1643	1702	60	2	
*trnD* (gac)	1703	1757	55	0	
*trnG* (gga)	1758	1812	55	0	
*COX2*	1813	2508	696	0	ATT/TAA
*trnH* (cac)	2512	2568	57	3	
*rrnL*	2569	3525	957	0	
*ND3*	3526	3861	336	0	ATT/TAA
*ND5*	3862	5445	1,584	0	ATT/TAA
*trnA* (gca)	5448	5502	55	2	
*trnP* (cca)	6199	6252	54	696	
*trnV* (gta)	6253	6306	54	0	
*ND6*	6307	6741	435	0	ATT/TAA
*ND4L*	6742	6975	234	0	ATT/TAA
*trnW* (tga)	6975	7031	57	–1	
*trnE* (gaa)	7037	7092	56	5	
*rrnS*	7093	7789	697	0	
*trnS2* (tca)	7788	7841	54	–2	
*trnN* (aac)	7842	7897	56	0	
*trnY* (tac)	7901	7957	57	3	
*ND1*	7955	8830	876	–3	TTG/TAA
*ATP6*	8860	9429	570	29	ATA/TAA
*trnK* (aaa)	9438	9499	62	8	
*trnL2* (tta)	9508	9563	56	8	
*trnS1* (aga)	9564	9619	56	0	
*ND2*	9620	10465	846	0	ATT/TAA
*trnI* (atc)	10466	10526	61	0	
*trnR* (cgt)	10528	10583	56	1	
*trnQ* (caa)	10584	10638	55	0	
*trnF* (ttc)	10647	10703	57	8	
*COB*	10704	11816	1,113	0	ATA/TAA
*trnL1* (cta)	11821	11875	55	4	
*COX3*	11876	12643	768	0	ATT/TAA
*trnT* (aca)	12654	12708	55	10	
*ND4*	12709	13941	1,233	126	TTG/TAA

The mitogenome gene arrangement is considered as a useful tool to interpret phylogenetic relationships (Boore and Brown, 1998; Fritzsch et al., 2006). In this study, we recovered a highly conserved gene order in Rhabditidae and Diplogasteridae. This is in line with their intimate sister relationships revealed by both rRNA and mitogenome base phylogeny (Holterman et al., 2006; Park et al., 2011; Kim et al., 2015). Consequently, gene order can be used as an informative reference in higher level phylogeny. Conversely, gene order varies greatly among many Enoplea nematode species, and even congeneric species may have completely different arrangements. Therefore, the use of gene order to infer phylogenetic relationship within Enoplea is limited (Tang and Hyman, 2007; Hyman et al., 2011).

Complete mitogenome has shown promise as an additional independently evolving genome for developing phylogenetic hypotheses for nematodes (Hu and Gasser, 2006; Sultana et al., 2013; Humphreys-Pereira and Elling 2014; Sun et al., 2014; Kern et al., 2020). Regardless of their importance, the study of nematode mitogenome falls far behind those of other metazoans (Ye et al., 2014). Compared with more than 25,000 described nematodes (Zhang, 2013), only around 200 species were sequenced for mitogenome (Kern et al., 2020). One main bottleneck that limits nematode mitogenome sequencing is the primer selection in long PCR amplification. Indeed, a success primer design is often challenging for using few but high variable existing references. As a result, plenty of primer pairs were designed for a specific species (Humphreys-Pereira and Elling, 2014; Sun et al., 2014; Kim et al., 2015). With the development of high-throughput sequencing techniques, assembly of mitogenome from whole genome sequencing data became a feasible alternative. However, only few species have been sequenced using this approach, e.g., *Globodera ellingtonae*, *Hoplolaimus columbus*, and *A. medicagus* (Phillips et al., 2016; Ma et al., 2020; Qing et al., 2021). Using the Illumina platform for sequencing and seed-extend algorithm assembly, we obtained the first mitogenome representative of the genus *Cruznema* and demonstrated that high-throughput sequencing is a promising option to acquire mitogenome in a time- and cost-efficient manner. Although substantially increased taxa sampling is still needed, our result updates mitogenome phylogeny and provides a valuable reference for future high-throughput sequencing based mitogenome research.
